# Utilization of overground exoskeleton gait training during inpatient rehabilitation: a descriptive analysis

**DOI:** 10.1186/s12984-023-01220-w

**Published:** 2023-08-04

**Authors:** Jaime Gillespie, Dannae Arnold, Molly Trammell, Monica Bennett, Christa Ochoa, Simon Driver, Librada Callender, Seema Sikka, Rosemary Dubiel, Chad Swank

**Affiliations:** 1 Baylor Scott and White Institute for Rehabilitation, 909 N. Washington Ave., Dallas, TX 75246 USA; 2 Baylor Scott and White Research Institute, 3434 Live Oak St., Dallas, TX 75204 USA; 3 Baylor Scott and White Research Institute, 909 N. Washington Ave., Dallas, TX 75246 USA; 4 Baylor Scott and White Research Institute and Baylor Scott and White Institute for Rehabilitation, 909 N. Washington Ave., Dallas, TX 75246 USA

**Keywords:** Walking, Robotic exoskeleton, Neurological rehabilitation, Stroke, Spinal cord injuries, Traumatic brain injury, Rehabilitation hospital, Physical therapy, Recovery of function

## Abstract

**Background:**

Overground exoskeleton gait training (OEGT) after neurological injury is safe, feasible, and may yield positive outcomes. However, no recommendations exist for initiation, progression, or termination of OEGT. This retrospective study highlights the clinical use and decision-making of OEGT within the physical therapy plan of care for patients after neurological injury during inpatient rehabilitation.

**Methods:**

The records of patients admitted to inpatient rehabilitation after stroke, spinal cord injury, or traumatic brain injury who participated in at least one OEGT session were retrospectively reviewed. Session details were analyzed to illustrate progress and included: “up” time, “walk” time, step count, device assistance required for limb swing, and therapist-determined settings. Surveys were completed by therapists responsible for OEGT sessions to illuminate clinical decision-making.

**Results:**

On average, patients demonstrated progressive tolerance for OEGT over successive sessions as shown by increasing time upright and walking, step count, and decreased assistance required by the exoskeleton. Therapists place preference on using OEGT with patients with more functional dependency and assess feedback from the patient and device to determine when to change settings. OEGT is terminated when other gait methods yield higher step repetitions or intensities, or to prepare for discharge.

**Conclusion:**

Our descriptive retrospective data suggests that patients after neurological injury may benefit from OEGT during inpatient rehabilitation. As no guidelines exist, therapists’ clinical decisions are currently based on a combination of knowledge of motor recovery and experience. Future efforts should aim to develop evidence-based recommendations to facilitate functional recovery after neurological injury by leveraging OEGT.

**Supplementary Information:**

The online version contains supplementary material available at 10.1186/s12984-023-01220-w.

## Background

The ability to regain walking function is a highly prioritized goal for individuals after neurological injury [1, 2] as well as rehabilitation clinicians.[3] Despite the emphasis placed on gait training during rehabilitation, recovery of independent community-level walking function is attained in only 37 to 60% post-stroke[4], and 73.3% post-traumatic brain injury (TBI) [5]. For those with spinal cord injury (SCI), recovery of independent community ambulation varies significantly with age and severity of injury resulting in 0–75% post-motor complete SCI, and 25 to 100% post-motor incomplete SCI [6]. Most often, individuals after neurologic injury live with disability due to residual deficits including imbalance, muscle weakness, increased fall risk, and greater energy expenditure [7] leading to barriers in resuming previous life roles [8, 9]. Even when these individuals regain some ability to walk, limitations in recovery often make walking as a primary method of mobility inefficient and impractical [10].

Consequently, new approaches to gait training are necessary following neurologic injury [11]. Due to advancing technology, overground exoskeleton gait training (OEGT) [7], is emerging as a feasible [12], safe [13], and potentially beneficial [14–16] intervention during inpatient rehabilitation. After stroke, current evidence suggests clinically significant improvements in gait outcomes with use of OEGT[17], with particular potential for improvement in the subacute phase [15]. Notably, patients with subacute stroke perceive walking in an exoskeleton as “enjoyable” and “comfortable” [17]. Similarly, gait deficits in those with TBI may resemble that of an individual who has experienced a stroke [18, 19] leading to the conclusion that OEGT may have a similar impact despite a different etiology of the acquired brain injury [19]. After SCI, findings report both physical (e.g., pain and spasticity reduction, improved strength, functional improvements) and psychological benefits with OEGT [20, 21].

Despite the potential benefits of OEGT for patients after neurological injury, there remain no established recommendations for initiation, progression, or termination of OEGT use during inpatient rehabilitation. Previous work has highlighted the need for strong clinical decision making skills by therapists to use OEGT [13]. For example, of four SCI Model Systems, only one center utilized OEGT during inpatient rehabilitation, and limited use to six sessions during the length of stay to allow adequate time for other rehabilitation interventions deemed necessary [20]. A recent protocol paper for a multi-site randomized controlled trial described detailed plans for progressing patients with subacute stroke with OEGT during inpatient rehabilitation [22]. While the subsequent study reported no differences between traditional therapy and an exoskeleton-based approach, the authors report providing “suggested training targets but allowed therapists to make their own clinical decisions” [23]. Importantly, the findings were determined to be partly attributable to the variability of the delivered OEGT intervention. As such, there is a pressing need for evidence-based recommendations to guide clinical practice for initiation, progression, and termination of OEGT [20].

This retrospective study highlights the clinical use and decision-making of OEGT within the physical therapy plan of care for patients after neurological injury during inpatient rehabilitation. Therapist commentary was obtained to supplement OEGT session data to underscore clinical decision making specific to patients with stroke, SCI, and TBI on the initiation, progression, and termination of OEGT during inpatient rehabilitation.

## Methods

### Participants

Patients were eligible if they completed a minimum of one OEGT session during inpatient rehabilitation following stroke, SCI, or TBI and were included in the medical record review. OEGT was completed with either the EksoGT [24] (original device) or EksoNR [25] robotic devices, which are wearable exoskeletons that allow full weight-bearing walking overground with the motor adaptability to allow the patient to move under their own control as much as possible [26, 27]. Whereas both devices have FDA class II approval for use with stroke, SCI, and TBI [28], the EksoNR is upgraded with advanced safety mechanisms, enhanced feedback on patient performance, and expanded options for treatment progression. To have participated in OEGT, patients were required to have met the following clinical and manufacturer criteria: (1) age greater than 18; (2) bowel and bladder program established or Foley catheter in place; (3) involved in standing program; (4) met the robotic exoskeleton frame limitations including weighing 100 kg or less, between 1.5 and 1.9 m tall, standing hip width of 0.45 m or less, near normal range of motion in hips, knees, and ankles, able to attain a neutral ankle dorsiflexion with < 12 degrees of knee flexion, no more than 12 degree hip flexion contracture, and no significant upper or lower leg length discrepancy. Patients were excluded from participating in OEGT for spinal instability, untreated deep vein thrombosis (DVT), decreased standing tolerance due to orthostatic hypotension, history of osteoporosis that increased the risk of fracture, uncontrolled spasticity, uncontrolled autonomic dysreflexia (AD), skin integrity issues on contact surfaces of the device, and pregnancy.

OEGT sessions included use of overground robotic exoskeletons only. Prior to the first OEGT session, the therapist completed measurements of the patient (i.e., limb length, hip width, and ranges of motion) to ensure optimal fit within the device. Subsequently, the first session incorporated familiarization to the device as OEGT was initiated. Other gait training approaches (i.e., overground with braces or treadmill walking) were not included in OEGT sessions.

### Procedure

Hospital institutional review board approval was obtained prior to initiating data collection. Medical records of patients admitted to an inpatient rehabilitation hospital within the United States between September 2016 and March 2022 were retrospectively reviewed. Within the United States healthcare model, patients are provided a minimum of three hours of therapy daily [29] over their inpatient rehabilitation length of stay (average of 16.9 days for stroke [30], 36 days for SCI [31], and 18.4 days for TBI [32]). At this specific rehabilitation hospital, physical therapy sessions are 45 min in length, and a patient may receive one to two physical therapy sessions daily. Once a list of participants who completed a minimum of one OEGT session was obtained, medical records were reviewed for stroke, SCI and TBI diagnoses by four trained data extractors. Oversight and auditing of data extraction was provided throughout the review process by a data analyst and included verification of the accuracy of each medical record and corresponding data entry form.

### Outcomes

Demographic data (age, gender, race, ethnicity, diagnosis, and inpatient rehabilitation length of stay) and diagnosis specific details were extracted for all included patients. For patients with stroke, side of lesion was extracted. For those with SCI, the ASIA Impairment Scale (AIS) was extracted for patients with traumatic injury and an AIS equivalent for non-traumatic injuries [33]. For patients with TBI, emergence from post-traumatic amnesia [34] was tracked to characterize severity of cognitive deficits.

Individual OEGT session data were extracted as captured by the exoskeleton device and included frequency of use and dose defined as “up” time (i.e., amount of time spent in the exoskeleton upright), “walk” time (i.e., amount of time spent walking in the exoskeleton), and number of steps. Device assistance details were captured to indicate how much the exoskeleton needed to assist each lower extremity during the swing phase of gait. These OEGT session details were analyzed across multiple sessions to illustrate any progress made in tolerability (i.e., longer up time, walk time, or greater number of steps per session) or device dependency (i.e., less assistance required by the device for swing). Additionally, therapist-determined device settings and changes were tracked across sessions to describe clinical decisions made to facilitate individual patient progress. Swing Assist Mode illustrated the amount of assistance provided by the device to complete the step trajectory (i.e., step length, step height, and swing time) during swing phase [25]. Swing Assist Mode was determined by the therapist during each session and options included Max, Adapt, Fixed, and Free [25] (characterized in Table 1). During each session, initial swing assist mode settings were documented and changes made during the session were noted as a “mid-session change”. Of note, swing assistance is only captured by the device in the Adapt and Fixed settings.

**Table 1:** Therapist-determined settings available for Swing Assist Mode with descriptors

Swing assist mode	Description
Max	Assistance is at 100% for step trajectory. Less susceptible to patient interaction
Adapt	The device can provide up to 100% assistance for step trajectory, but only provides this assistance if needed. Constantly adjusts to patient power needs
Fixed	The ceiling amount of assistance is set to a fixed number (100–0), as determined by the clinician. The patient may use *up to*, or *less than*, the programmed value to complete swing along the trajectory
Free	The limb is *not* being controlled in a trajectory pattern. Allows the patient more freedom to control the step, but the device can provide powered assistance or resistance through the step, as programmed by the therapist

“Step trajectory” is the path and speed of the step and is a combination of step height, step length, and swing time [25]

### Therapist commentary

Following data review, surveys were completed by the therapists responsible for planning and executing the OEGT sessions. Survey items (provided in Additional file 1: Appendix S1) were designed to illuminate the clinical decision-making process involved in the initiation (e.g., patient selection, determining patient readiness), progression (e.g., device setting selection), and termination (e.g., discontinuation in favor of alternative gait interventions) of OEGT during inpatient rehabilitation.

### Data analysis

Demographic and injury-related characteristics were summarized using means and standard deviations or medians and interquartile ranges for continuous variables, and counts and percentages for categorical variables. Demographic variables were summarized overall and stratified by diagnosis group. In an effort to avoid skewing data due to low sample size at higher number of sessions, OEGT session metrics were summarized for sessions in which approximately 75 percent of the sample had data for each impairment group, yielding up to 8 sessions for patients with stroke, 9 for SCI, and 12 for TBI. Continuous session metrics were summarized with means and standard deviations, and categorical metrics were summarized with counts and percentages. Bar graphs and line graphs were used to visualize the trends and change in session metrics over time. All analysis was performed using SAS 9.4 (SAS Institute, Cary, NC).

## Results

### Demographic and injury characteristics

Of 3,912 (1,546 stroke, 981 SCI, and 1,385 TBI) admissions, there were 228 patients included in this analysis: 104 stroke, 99 SCI, and 25 TBI (Table 2). Patients with stroke were the oldest with an average age of 55.2 years and patients with TBI were the youngest with an average age of 38.3 years. All patient groups were majority male (67%) and identified as white race (67%). Approximately 39% of patients with stroke were right-sided and 37% were left-sided. Over half (56%) of patients with SCI had cervical level injury, and 47% had motor incomplete impairment based on AIS (and equivalent) scores. For patients with TBI, 40% emerged from post-traumatic amnesia by inpatient rehabilitation discharge. Median length of stay was 35 days for those admitted with stroke, 42 days for SCI, and 40 days for TBI.

**Table 2:** Demographic and clinical characteristics

	Overall (n = 228)	SCI (n = 99)	Stroke (n = 104)	TBI (n = 25)
Age at injury, years (mean ± std. dev.)	48.5 ± 17.5	44 ± 17.8	55.2 ± 13.9	38.3 ± 19.5
Injury to first OEGT, days (mean ± std. dev.)	62 ± 169.1	95.3 ± 219.7	29.6 ± 111	61.8 ± 94
Height, centimeters (mean ± std. dev.)	173 ± 8.9	173.7 ± 9.7	171.5 ± 8.1	175.5 ± 8.6
Weight, kilograms (mean ± std. dev.)	75.8 ± 15.1	74.7 ± 15.0	76.1 ± 16.0	69.9 ± 10.8
BMI (mean ± std. dev.)	25 ± 4.4	24.7 ± 4.3	25.8 ± 4.5	22.8 ± 3.7
Male Sex	153 (67.1%)	72 (72.7%)	62 (59.6%)	19 (76%)
Race				
White	152 (66.7%)	66 (66.7%)	67 (64.4%)	19 (76%)
African American/Black	43 (18.9%)	16 (16.2%)	24 (23.1%)	3 (12%)
Hispanic	12 (5.3%)	6 (6.1%)	5 (4.8%)	1 (4%)
Other	21 (9.2%)	11 (11.1%)	8 (7.7%)	2 (8%)
SCI level				
Cervical		55 (55.6%)		
Thoracic		34 (34.3%)		
Lumbar		5 (5.1%)		
Missing/Unknown		3 (3%)		
ASIA impairment scale				
A		12 (12.1%)		
B		14 (14.1%)		
C		23 (23.2%)		
D		24 (24.2%)		
Missing/Unknown		26 (26.3%)		
Stroke type				
Left			38 (36.5%)	
Right			40 (38.5%)	
Bilateral			9 (8.7%)	
Missing/Unknown			17 (16.3%)	
Emerged from PTA				
No				11 (44%)
Yes				10 (40%)
Unknown/Missing				4 (16%)
FIM–motor total	n = 110	n = 41	n = 59	n = 10
Admission	23.7 ± 10.1	22.2 ± 11.0	25.6 ± 9.5	17.7 ± 6.2
Discharge	49.9 ± 15.7	51.4 ± 18.0	49.9 ± 13.7	43.9 ± 16.8
Change	26.3 ± 12.2	29.1 ± 14.9	24.3 ± 9.4	26.2 ± 13.9
CARE–total	n = 113	n = 54	n = 44	n = 15
Admission	10.4 ± 4.5	11.7 ± 5.2	9.8 ± 3.5	7.7 ± 1.3
Discharge	27 ± 13.6	30.9 ± 14.2	23.4 ± 11.7	20.0 ± 11.4
Change	16.4 ± 12.9	19.4 ± 14.5	13.4 ± 9.9	12.2 ± 11.3

*PTA* post-traumatic amnesia, *std dev* standard deviation, *BMI* body mass index, *SCI* spinal cord injury, *TBI* traumatic brain injury, *OEGT* overground exoskeleton gait training, *FIM* Functional Independence Measure (used to document functional dependency prior to October 1, 2019); *CARE* Continuity Assessment Record and Evaluation (used to document functional dependency after October 1, 2019)

### Session counts

Patients with TBI averaged the highest number of total OEGT sessions (9.1 ± 5.6) and sessions per week during inpatient rehabilitation (1.73 ± 1.44). Patients with stroke and patients with SCI had similar OEGT session counts (6.3 ± 4.1 and 5.8 ± 4.4, respectively) and average number of sessions per week (1.05 ± 0.65 and 1.17 ± 0.74, respectively).

### Up time, walk time, and step count

**Fig. 1:**
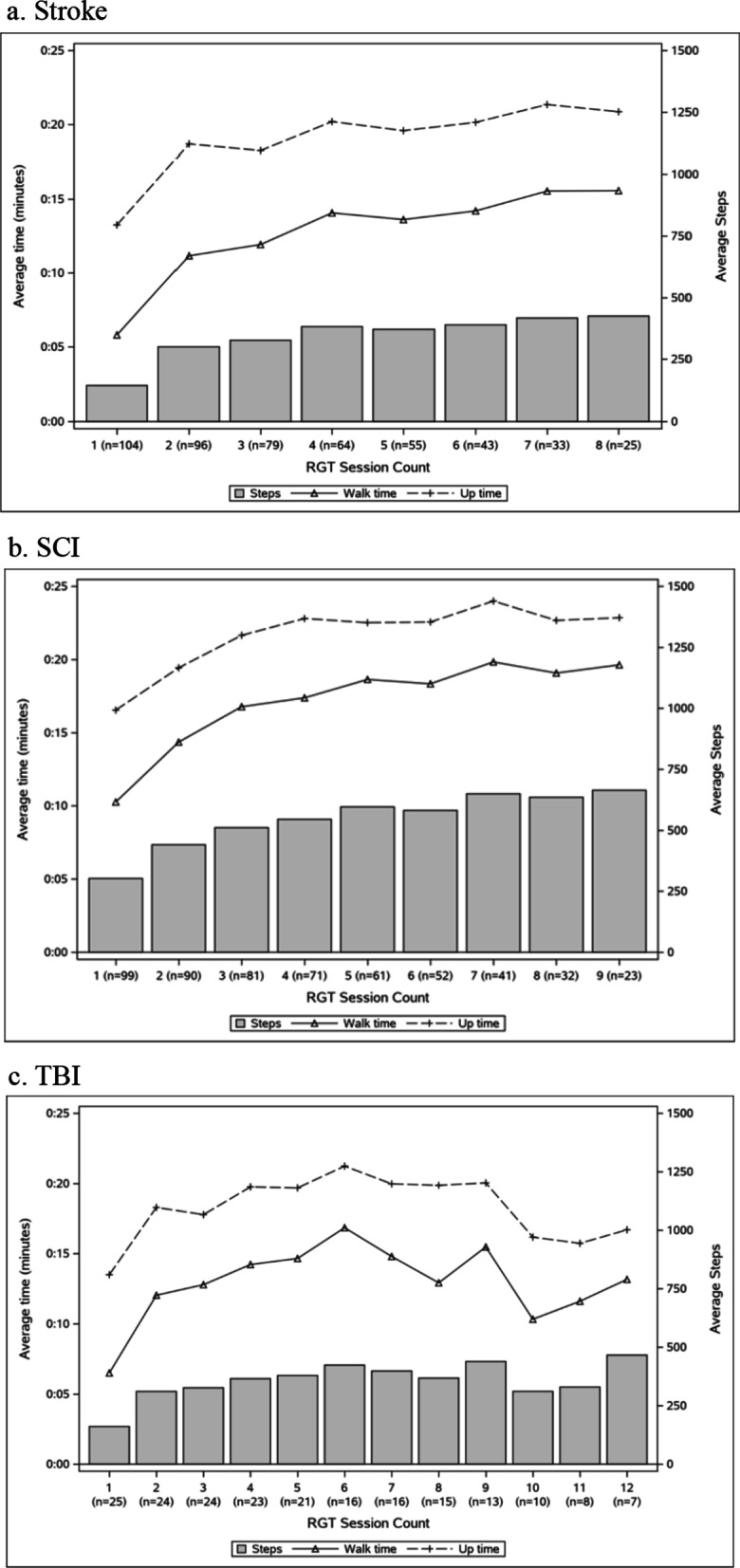
OEGT session up time, walk time, and step counts over multiple sessions for each diagnosis group. Detailed means and standard deviations provided in Additional file 2: Appendix S2

**Fig. 2:**
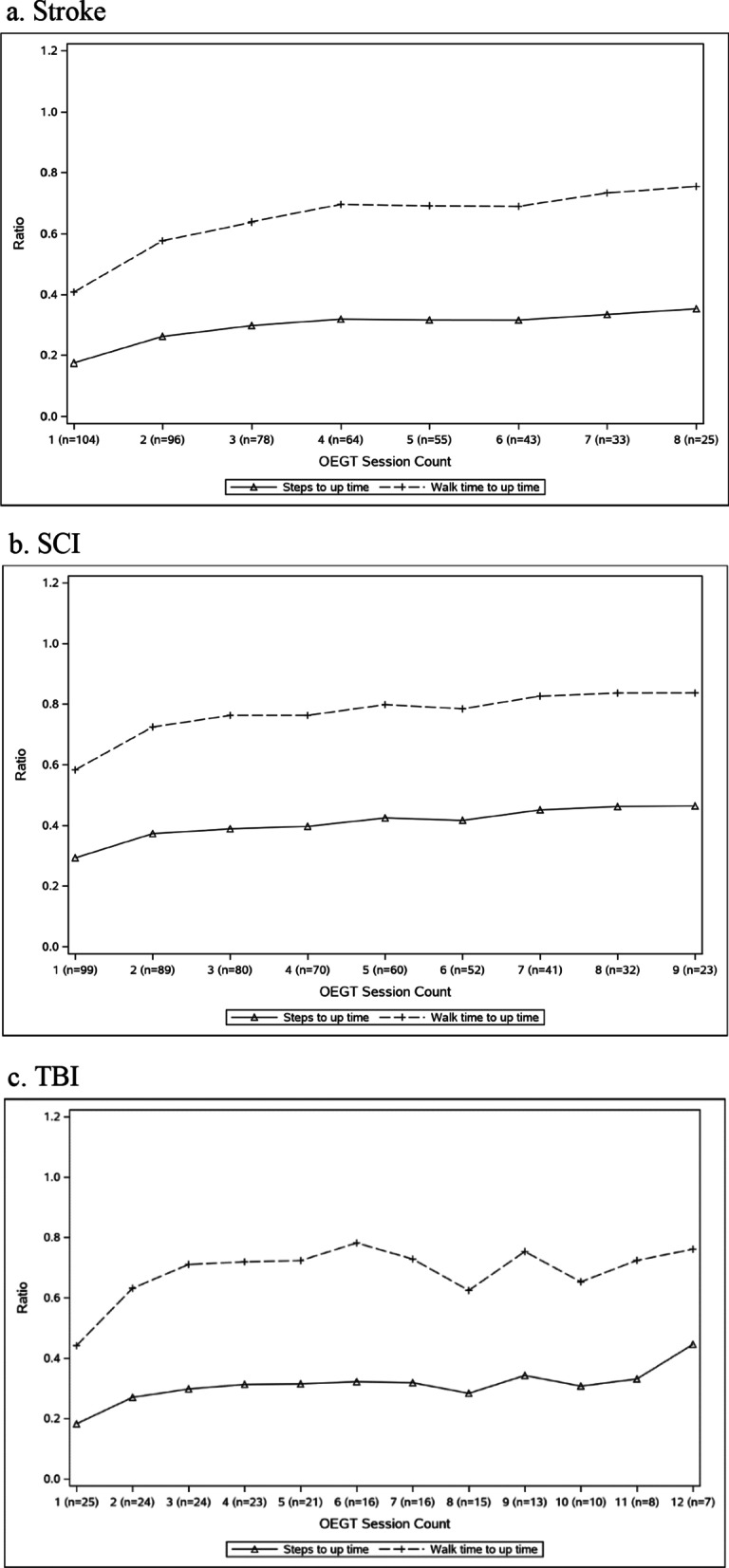
Ratios of walk time to up time and number of steps to up time for each diagnosis group

OEGT session up time, walk time, and step counts are summarized in Fig. 1 and detailed means and standard deviations provided in Additional file 2: Appendix S2. The ratio of walk time to up time and number of steps to up time (summarized in Fig. 2 and Additional file 2: Appendix S2) generally increased over multiple sessions for stroke, SCI, and TBI. For all diagnosis groups, the largest single session increase was observed between sessions 1 and 2, with steady improvement continuing over most subsequent sessions. The average walk time and step count for patients with stroke tripled from the first session (walk time = 5:48 min; step count = 143) to the eighth session (walk time = 17:16 min; step count = 440). Patients with SCI averaged the most up time (16:34 min), walk time (10:15 min), and step count (303) in the first session and continued to tolerate increases by doubling the walk time to 20:08 min and average step count to 671 by session 9. Patients with TBI had the most variability over time in up time, walk time, and step count across sessions, but still showed overall improvement from session 1 (up time = 13:30 min; walk time = 6:30; steps = 161) to session 12 (up time = 16:42; walk time = 13:10; step count = 466).

### Device assistance for swing phase

**Fig. 3:**
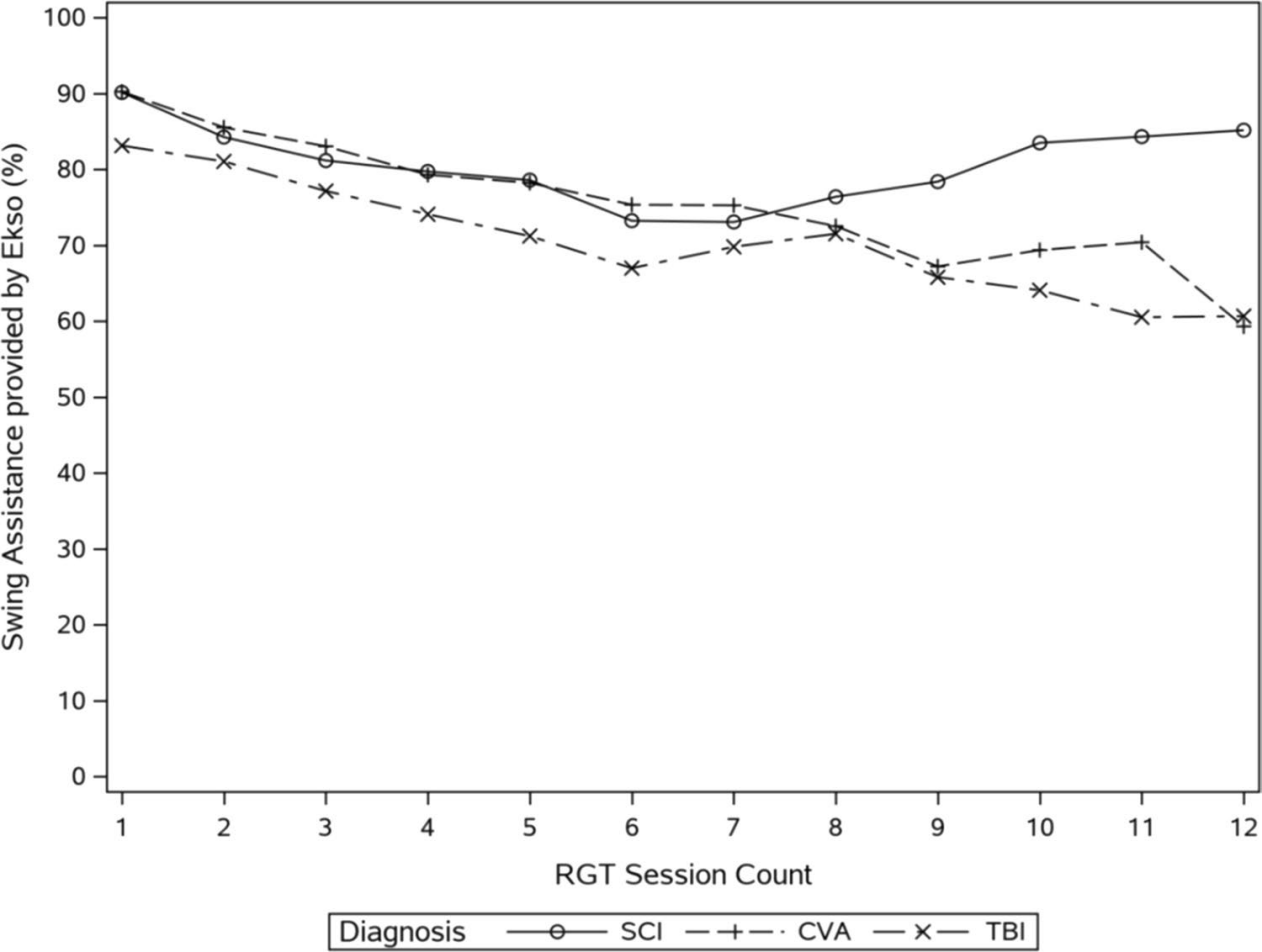
Mean device assistance required for swing phase of gait across multiple sessions for each diagnosis

Mean OEGT session swing assistance values are provided in Fig. 3. In general, device assistance for swing decreased across sessions for patients with stroke, SCI, and TBI. However, swing assistance was observed to increase for the patients with SCI who received more than seven sessions. By contrast, a steady decline of swing assistance to approximately 60% was observed for patients with stroke and TBI.

### Swing Assist Mode with mid-session changes

**Fig. 4:**
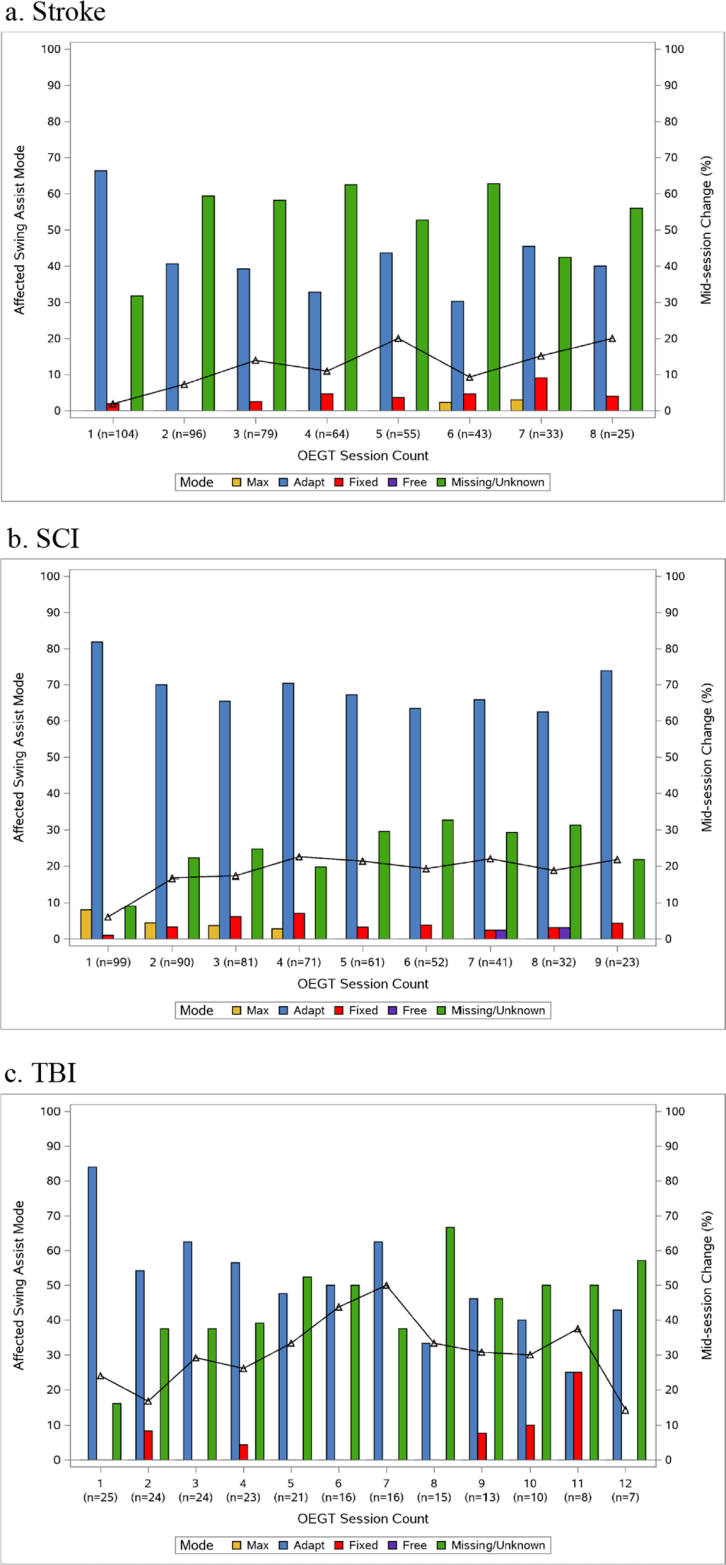
Therapist-determined Swing Assist Modes for each diagnosis group

Therapist-determined Swing Assist Modes are displayed in Fig. 4. Adapt was the most selected mode at session initiation for all diagnoses groups. Additionally, mid-session setting changes increased in frequency across sessions for stroke (1.9 to 20%), SCI (6 to 25%) and TBI (15 to 50%).

### Therapist commentary

**Fig. 5:**
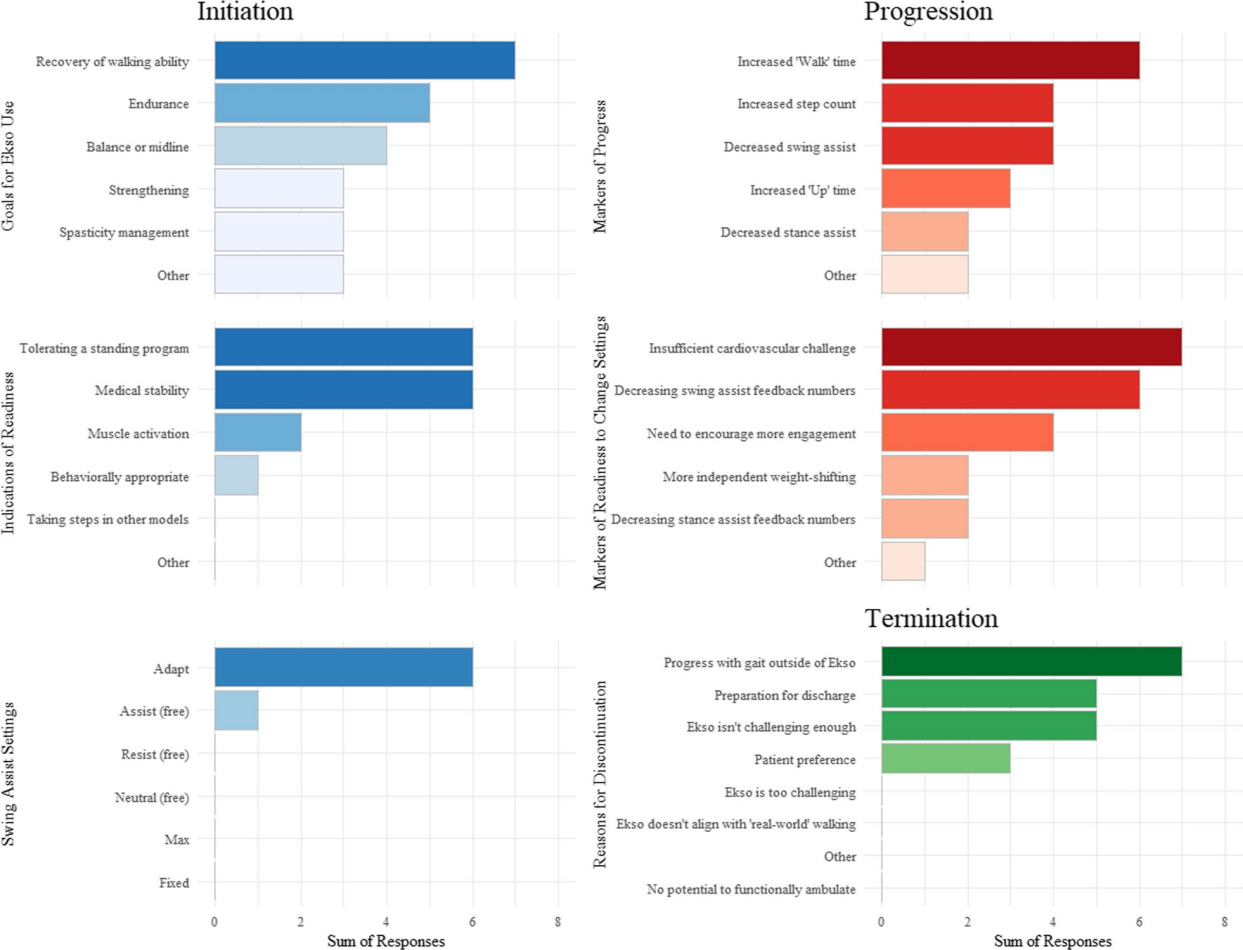
Physical therapists’ responses to multiple choice survey questions designed to describe clinical decisions informing the initiation, progression, and termination of OEGT during inpatient rehabilitation for patients after stroke, SCI, or TBI. See Additional file 1: Appendix S1 for survey questions and response options. Seven therapists were surveyed and were instructed to ‘mark all that apply’. Higher number of responses indicate more therapists chose the specific response

Seven therapists participated in a semi-structured survey to provide insight for OEGT clinical decision-making (see Additional file 1: Appendix S1). All therapists had completed training for use of Ekso Bionics robotic exoskeleton devices. Overall, therapists had 8.9 years of clinical experience and 4.5 years of experience with OEGT. Therapists specialized in stroke (n = 2), SCI (n = 2), TBI (n = 2), and general debility (n = 1). Responses to multiple choice survey questions are illustrated in Fig. 5 and comments specific to clinical decision making for initiation, progression, or termination of OEGT are presented in Table 3.

**Table 3:** Physical therapists’ comments specific to clinical decisions for OEGT after neurological injury during inpatient rehabilitation

Topic	Therapist commentary
Initiation	*“I use it for patients that would be impossible to get up and walking any other way.”* *“To me, Ekso is the most controlled/supportive form of gait training requiring the least amount of stress on both patient and staff. I often have patients ready for this before anything else.”* *“The determination for Ekso to be the primary gait training device is dependent on quality of steps, the amount of intensity, and the repetition of steps as compared to other gait training environments.”*
Progression	*“As soon as I see a decrease in swing assistance or their vitals aren't being challenged I will drive all numbers down in Fixed for less assistance from Ekso as soon as I can because the point of my Ekso treatments is to get them walking over ground.”* *“As heart rate decreases in response to workload, I am clued in that they ready for more load. So, I decrease assist or add resistance and observe quality. If they are constantly corrected by the machine, I increase assist levels back to where they are working hard but successful most of the time.”*
Termination	*“Typically, when a patient gets to Free I move to over ground walking outside of the Ekso so I can incorporate multi-directional walking, stair training, changes in speed, *etc*.”* *“For CVA or TBI, once they understand the concept of midline, gain some trunk control, and learn the general requirements to produce a reciprocal pattern, I will begin incorporating overground training.”* *“If the patient progresses to the point where gait overground or within another device provides more repetition, intensity, and variability then I will transition them out of the device.”*

## Discussion

The purpose of this retrospective study was to highlight the clinical use and decision-making of OEGT within the physical therapy plan of care for patients after neurological injury during inpatient rehabilitation. On average, patients with stroke, SCI, and TBI demonstrated progressive tolerance for OEGT as demonstrated by increasing time upright, time walking, and number of steps over successive sessions. Further, the amount of assistance provided by the robotic exoskeleton generally decreased across those same sessions. Therapist commentary was used to illuminate clinical decision-making that that contributed to the quantitative trends observed in this retrospective analysis. Reflection, evaluation, and making reactive changes are critical to the successful implementation of a new intervention and evolution of clinical practice [35]. As such, the therapists’ perspectives are the product of lessons learned over 6 years.

### Initiation of OEGT

Prior to initiating OEGT, patients must demonstrate standing tolerance, largely to ensure that orthostatic hypotension [36] is less likely to occur as the patient is walking in an exoskeleton. Perhaps an intuitive observation, nonetheless, manufacturer training guidelines do not specify how to determine standing tolerance. All therapists surveyed indicated “tolerating a standing program” as a primary milestone prior to beginning OEGT. However, the therapists noted an observed greater tolerance for being upright during OEGT compared to a passive standing program in a standing frame. Potentially, the stepping during OEGT allowed for improved venous return in those with neurogenic orthostatic hypotension compared to static standing [37, 38]. Early adoption of OEGT during inpatient rehabilitation could afford patients the opportunity to participate in walking retraining sooner than other gait training methods and therefore maximize the limited time available during inpatient rehabilitation [39].

Moreover, our therapists described initiating OEGT prior to other gait training interventions (e.g., body weight supported treadmill or overground walking) for patients who were more severely impaired. Specifically, therapists preferred OEGT for patients “that would be impossible to walk any other way” because of the “supportive” and “controlled” nature of OEGT. Walking interventions using OEGT were described as “less burdensome” (i.e., less physical demand on the therapist or less staff required) and “more successful” (i.e., higher repetition of steps). Contemporary neurorehabilitation recommendations highlight the importance of providing mobility training even for those with severe mobility impairments [40] and OEGT may allow for this opportunity earlier than other gait training methods and with less burden on the therapist [20] due to the supportive but adaptable nature of OEGT compared to more traditional gait training methods.

### Progression of OEGT

Having initiated OEGT, therapists deliberately sought to progress OEGT to promote recovery of function. Progression of OEGT was achieved by increasing the time walking and step count and reducing device assistance. Further, the ratio of time walking and step count of our patients to up time trended higher as OEGT sessions progressed with the greatest increase for each diagnosis group between sessions one and two. However, the ratio of walk time to up time plateaued around 0.8 suggesting the necessity of time spent standing but not walking (e.g., checking vital signs, assessing alignment of the device, standing rest breaks) amidst progressing gait training efforts.

Therapists reported patients were ready to progress when individual sessions yielded (1) decreasing assistance needed during the swing phase of gait as indicated by device feedback numbers and (2) therapist-monitored cardiovascular intensity was low despite increased session time and steps. It is human instinct to take advantage of sources for reducing energy expenditure during walking [41] (e.g., the robotic motors) and therapists must be intentional to challenge patients during OEGT sessions. Device feedback monitored by the therapists during OEGT sessions allowed for continuous opportunities to challenge the patient by adopting mid-session changes to reduce swing assistance levels. Specifically, therapists utilized a technique referred to as “submarining” to drop the fixed assist value just below the Swing Assist feedback value. This approach encourages patients to strive to achieve lower fixed assist values for continual challenge. This submarine method continues until the patient approaches relatively low fixed assist values, whereby the therapists consider transition from Fixed to Free mode, to allow the lower limb to not be controlled in the device step trajectory pattern. This method of reducing device assistance has been previously described [22, 42] to provide progressive challenge to the patient during OEGT. However, contemporary robotic exoskeleton devices do not mimic a natural gait pattern in healthy adults [43]. As such, future studies should consider allowing trajectory-free stepping earlier in attempt to promote motor relearning by allowing patients to have more autonomy over their stepping pattern.

Patients in all diagnoses generally demonstrated reduced assistance from the device for swing phase as session count progressed, except for those with SCI who demonstrated an uptick in assistance required from the device after session seven. This observed increase in assistance in later sessions is possibly due to those with more severe injuries (and less potential to participate in swing phase) having longer lengths of stay [44] and therefore more opportunities for higher session counts. Additionally, given that swing assistance was only recorded by the device in Adapt and Fixed settings, it is possible a lack of swing assistance recorded during later OEGT sessions utilizing Free settings influenced our findings. Nonetheless, differences in pathology among patients with stroke, SCI, and TBI will likely yield varying treatment progressions during OEGT. For example, progression for a patient with hemiparesis after stroke may include unilateral assistance only or a patient with less severe (e.g., AIS D SCI) injuries may incorporate variable stepping [14].

Therapists also noted the need to progress OEGT settings when the cardiovascular intensity elicited by the current settings was low, e.g., less than 60% of heart rate reserve. Recent literature has emphasized the importance of ensuring sufficient cardiovascular intensity to enhance motor recovery after neurological injury [45, 46]. Others have encouraged reflection after each OEGT session to determine appropriate setting changes to meet cardiovascular intensities recommended during gait training [47].

### Termination of OEGT

No evidence or manufacturer suggestions currently exist to support clinical decision-making regarding termination of OEGT. Although many patients continued OEGT throughout their entire inpatient rehabilitation stay, termination of OEGT most often occurred to “progress with gait outside of OEGT”. Therapists transitioned away from OEGT to select a gait training modality once they observed a plateau of therapy session intensity and repetition [39, 48]. For patients with stroke or TBI, therapists emphasized the importance of regaining “trunk control” within “midline” prior to transitioning to overground walking. Therapists described gait outside of OEGT as “generalizing more quickly”, possibly because OEGT does not mimic normal gait patterns [43] and walking in variable environments (e.g., stair and obstacle negotiation, multi-directional walking) may more readily adapt patients to walking in real-world settings [49, 50].

Another common reason for termination of OEGT was to allow time for discharge preparation, including training of caregivers. Despite walking being a highly prioritized goal after neurological injury [1, 2], independence with walking is not a requirement prior to discharge from inpatient rehabilitation. Importantly, providing caregiver training is associated with higher satisfaction of care [51] and can improve physical, mental, and emotional outcomes for both the patient and the caregiver [52]. Thus, balancing competing priorities within the constraints of an inpatient rehabilitation stay often requires forgoing task-specific gait training [39, 48] (with the aim of maximizing motor recovery) for caregiver education (with the aim at achieving a safe discharge home).

Some (8 stroke, 10 SCI, and 1 TBI) patients completed only one OEGT session during their inpatient rehabilitation length of stay. Consistent with others, reasons for a single session included therapist prioritization of other interventions to improve functional independence (e.g., wheelchair propulsion and transfers) [20], abbreviated inpatient rehabilitation length of stay [13], and periodically reported fearfulness or anxiety during the initial session preventing continue participation [13]. However, most patients were willing and able to participate in a second OEGT session [53].

### Next steps

Given our findings, we suggest specific recommendations as next steps to expand the knowledge base guiding clinical use and decision-making of OEGT within the physical therapy plan of care during inpatient rehabilitation.

A) Initiation: Prospective examination of patients’ tolerance for dynamic upright positioning with OEGT compared to passive standing in a standing frame. A favorable outcome may allow for earlier initiation of OEGT and task-specific training.
B) Initiation: Determination of the physiological burden on therapists during OEGT compared to usual care gait training interventions (e.g., body weight supported treadmill or overground walking). Our therapists perceived OEGT to be less burdensome which influenced their use of exoskeletons for mobility training.
C) Progression: Incorporation of cardiovascular monitoring during OEGT for patients with neurological injuries. In response to recent literature emphasizing the importance of interventions eliciting sufficient cardiovascular intensity [46], our therapists highlighted the need to adjust OEGT settings when the cardiovascular intensity was low. However, understanding the capacity to elicit cardiovascular intensity through specific OEGT settings may be vital to progressing the intervention [47]. Prior studies suggest OEGT may be capable of eliciting cardiovascular responses into moderate and vigorous ranges during inpatient rehabilitation for patients with stroke, SCI, and TBI [11].
D) Progression: Modification of manufacturer provided OEGT data collection forms to prompt notation of details of mid-session setting changes. Contemporary exoskeletons offer expanded software options for individualizing settings [54]. However, manufacturer provided documentation templates used by our therapists did not account for the potential of mid-session OEGT setting changes. So, while our therapists attempted to record session changes, the documentation template did not prompt such notation. Thus, specific settings of mid-session changes may not have been documented within the medical record or available for retrospective review. Capturing this data will allow for greater characterization of therapy progression and associated outcomes.
E) Termination: Establishment of functional milestones for termination of OEGT. Reasons provided by our therapists for termination of OEGT appeared to be based primarily upon device limitations and environmental considerations (e.g., timing of discharge).

### Study limitations

This retrospective study has several limitations which restrict our ability to generalize findings. First, our review of OEGT sessions was limited to the data in the medical record as documented at the time the patients received care. For example, much of device and session data (i.e., OEGT device settings and mid-session changes) were missing from the medical record, leading to difficulty drawing concrete conclusions. Further, specific device settings and their association with swing assist values were not tracked in the medical record (e.g., stance support required from device, setting differences between affected and less affected lower extremities), limiting the ability to gauge their contribution to our outcomes. Second, a lack of control subjects (or comparable data tracked during traditional gait training such as step count or time spent walking) within this review contributed to the inability to base clinical decisions on this data alone. However, the inclusion of therapist perspective may have added nuance to understanding the data. Limited sample size for patients with TBI further limits generalizability of this specific data. Additionally, policy changes in the United States healthcare system in 2019 lead to a change in required functional outcome measures. Consequently, the medical record reviewed for this study did not have a consistent functional description of patients participating in OEGT limiting the ability to correlate OEGT status with functional metrics. Lastly, all OEGT sessions were completed using devices from a single manufacturer. Other FDA approved robotic exoskeleton devices may have different device settings and utilization capacities for inpatient rehabilitation populations.

## Conclusion

Retrospective data from OEGT sessions for patients after neurological injury during inpatient rehabilitation indicates progressive tolerance for session duration with less device assistance required. Therapists articulated clinical benchmarks to initiate, progress, and terminate OEGT sessions for patients with stroke, SCI, and TBI. Specifically, clinical benchmarks supported early adoption and tailored progression of OEGT and deliberate transition to real-world walking approaches. Although additional clarity is needed to define optimal clinical practice of OEGT, particularly around progression of OEGT sessions, this descriptive retrospective analysis provides recommendations on clinical application of OEGT during inpatient rehabilitation.

### Supplementary Information

**Additional file 1. Appendix S1.** Clinical decision-making survey completed by the therapists responsible for planning and executing the OEGT sessions.

**Additional file 2. Appendix S2.** Means and standard deviations to supplement Fig. 1 (OEGT session up time, walk time, and step counts over multiple sessions for each diagnosis group) and Fig. 2 (ratios of walk time to up time and number of steps to up time for each diagnosis group)

## Data Availability

The data set used and/or analyzed during the current study are available from the corresponding author on reasonable request.

## Abbreviations

OEGT: Overground exoskeleton gait training
SCI: Spinal cord injury
TBI: Traumatic brain injury
AIS: ASIA impairment scale
