# Supplementary material for: Utilization of overground exoskeleton gait training during inpatient rehabilitation: a descriptive analysis
Source: J Neuroeng Rehabil. 2023 Aug 4;20:102. doi: 10.1186/s12984-023-01220-w (PMC10401799; doi:10.1186/s12984-023-01220-w)
Supplement: Supplementary file 1 — Additional file 1. Appendix S1. Clinical decision-making survey completed by the therapists responsible for planning and executing the OEGT sessions. [file 12984_2023_1220_MOESM1_ESM.docx]

| Appendix 1. Clinical decision-making survey completed by the therapists responsible for planning and executing the OEGT sessions. | |
| --- | --- |
| **Tell us about yourself** | |
| What population do you have the most experience working with in OEGT? | Stroke  SCI  TBI  Other: |
| How many years of experience do you have as a physical therapist? |  |
| How many years do you have as an OEGT trained clinician? |  |
| **Initiation of OEGT** | |
| Most frequently for your population, what are the goals for using OEGT? (mark all that apply) | Recovery of walking ability  Balance or midline  Spasticity management  Endurance  Strengthening  Other: |
| Comment on OEGT goals specific to your population. |  |
| Specific to your population, what factors indicate a patient is ready to begin OEGT gait training? (mark all that apply) | Medical stability  Tolerating a standing program  Already taking steps in other modes (e.g, BWSTT)  Demonstrating muscle activation  Behaviorally appropriate (e.g., absent/low agitation or impulsivity)  Other: |
| Comment on OEGT readiness specific to your population. |  |
| Why might you choose OEGT over other walking interventions? |  |
| What initial swing assistance device settings do you typically choose for your population? |  |
| Comment on the initial settings you choose with your population. |  |
| **Progression of OEGT** | |
| What “progress” most aligns with the goals you have for your patients when using the OEGT? (mark all that apply) | Increased “up” time  Increased “walk” time  Increased step count  Decreased swing assist  Decreased stance assist  Other: |
| What indicates a patient is ready for changes to OEGT settings? | Feedback numbers show decreasing swing assist  Feedback numbers show decreasing stance assist  They are weight-shifting more independently  Not enough cardiovascular challenge  Need to encourage more engagement  Other: |
| Comment on readiness for progression of settings. |  |
| **Termination of OEGT** |  |
| Most commonly for your population, why would you discontinue use of the OEGT? | They don’t have potential to functionally ambulate  Patient preference  OEGT isn’t challenging enough  OEGT is too challenging  OEGT doesn’t align with “real world” walking  Progress with gait outside of OEGT  Preparation for discharge (family training, equipment, etc)  Other: |
| Comment on the clinical decision making related to transitioning away from OEGT. |  |
