# Supplementary material for: Utilization of overground exoskeleton gait training during inpatient rehabilitation: a descriptive analysis
Source: J Neuroeng Rehabil. 2023 Aug 4;20:102. doi: 10.1186/s12984-023-01220-w (PMC10401799; doi:10.1186/s12984-023-01220-w)
Supplement: Supplementary file 2 — Additional file 2. Appendix S2. Means and standard deviations to supplement Fig. 1 (OEGT session up time, walk time, and step counts over multiple sessions for each diagnosis group) and Fig. 2 (ratios of walk time to up time and number of steps to up time for each diagnosis group) [file 12984_2023_1220_MOESM2_ESM.docx]

Appendix 2. Means and standard deviations to supplement Figure 1 (OEGT session up time, walk time, and step counts over multiple sessions for each diagnosis group) and Figure 2 (ratios of walk time to up time and number of steps to up time for each diagnosis group)

**Stroke**

|  | **Session Count** | | | | | | | |
| --- | --- | --- | --- | --- | --- | --- | --- | --- |
|  | 1 | 2 | 3 | 4 | 5 | 6 | 7 | 8 |
| **Walk time** |  |  |  |  |  |  |  |  |
| n | 104 | 96 | 78 | 64 | 55 | 43 | 33 | 25 |
| mean ± sd | 5:48 ± 4:23 | 11:10 ± 5:44 | 11:55 ± 5:23 | 14:04 ± 5:14 | 13:37 ± 5:38 | 14:12 ± 5:26 | 15:32 ± 5:16 | 15:34 ± 5:15 |
| **Up Time** |  |  |  |  |  |  |  |  |
| n | 104 | 96 | 78 | 64 | 55 | 43 | 33 | 25 |
| mean ± sd | 13:15 ± 7:50 | 18:41 ± 7:16 | 18:14 ± 7:44 | 20:11 ± 5:49 | 19:35 ± 6:04 | 20:08 ± 6:04 | 21:21 ± 7:05 | 20:51 ± 7:10 |
| **Steps** |  |  |  |  |  |  |  |  |
| n | 104 | 96 | 78 | 64 | 55 | 43 | 33 | 25 |
| mean ± sd | 143.3 ± 116.4 | 300.2 ± 167.9 | 326.8 ± 164.2 | 382.3 ± 166.4 | 371.2 ± 177 | 389.4 ± 174.2 | 417.1 ± 161.7 | 425.1 ± 142.8 |
| **Walk Time to Up Time Ratio** |  |  |  |  |  |  |  |  |
| n | 104 | 96 | 78 | 64 | 55 | 43 | 33 | 25 |
| mean ± sd | 0.43 ± 0.3 | 0.72 ± 1.42 | 0.72 ± 0.79 | 0.69 ± 0.18 | 0.69 ± 0.16 | 0.69 ± 0.16 | 0.73 ± 0.16 | 0.75 ± 0.1 |
| **Steps to Up Time Ratio** |  |  |  |  |  |  |  |  |
| n | 104 | 96 | 78 | 64 | 55 | 43 | 33 | 25 |
| mean ± sd | 0.17 ± 0.11 | 0.34 ± 0.78 | 0.33 ± 0.3 | 0.32 ± 0.11 | 0.32 ± 0.11 | 0.32 ± 0.11 | 0.33 ± 0.12 | 0.35 ± 0.09 |

**SCI**

|  | **Session Count** | | | | | | | | |
| --- | --- | --- | --- | --- | --- | --- | --- | --- | --- |
|  | **1** | **2** | **3** | **4** | **5** | **6** | **7** | **8** | **9** |
| **Walk time** |  |  |  |  |  |  |  |  |  |
| n | 99 | 89 | 80 | 70 | 60 | 52 | 41 | 32 | 23 |
| mean ± sd | 10:16 ± 5:26 | 14:21 ± 5:55 | 16:46 ± 6:25 | 17:23 ± 5:47 | 18:38 ± 6:28 | 18:20 ± 6:20 | 19:50 ± 5:34 | 19:04 ± 5:18 | 19:38 ± 7:09 |
| **Up Time** |  |  |  |  |  |  |  |  |  |
| n | 99 | 89 | 80 | 70 | 60 | 52 | 41 | 32 | 23 |
| mean ± sd | 16:34 ± 6:12 | 19:26 ± 6:27 | 21:39 ± 6:09 | 22:48 ± 6:22 | 22:31 ± 7:11 | 22:33 ± 6:20 | 23:59 ± 6:12 | 22:40 ± 5:07 | 22:51 ± 7:02 |
| **Steps** |  |  |  |  |  |  |  |  |  |
| n | 99 | 90 | 80 | 70 | 59 | 52 | 41 | 32 | 23 |
| mean ± sd | 302.5 ± 176 | 440.6 ± 194.9 | 510.6 ± 210.4 | 544.9 ± 219.2 | 595.8 ± 230.5 | 581.4 ± 240.7 | 649.5 ± 228.4 | 635.3 ± 232.5 | 663.8 ± 288.9 |
| **Walk Time to Up Time Ratio** |  |  |  |  |  |  |  |  |  |
| n | 99 | 89 | 80 | 70 | 60 | 52 | 41 | 32 | 23 |
| mean ± sd | 0.64 ± 0.6 | 0.73 ± 0.18 | 0.76 ± 0.16 | 0.76 ± 0.15 | 1.03 ± 1.85 | 0.8 ± 0.2 | 0.83 ± 0.1 | 0.84 ± 0.12 | 0.84 ± 0.12 |
| **Steps to Up Time Ratio** |  |  |  |  |  |  |  |  |  |
| n | 99 | 89 | 80 | 70 | 59 | 52 | 41 | 32 | 23 |
| mean ± sd | 0.29 ± 0.14 | 0.37 ± 0.13 | 0.39 ± 0.11 | 0.4 ± 0.11 | 0.52 ± 0.74 | 0.42 ± 0.13 | 0.45 ± 0.1 | 0.46 ± 0.12 | 0.46 ± 0.11 |

sd = standard deviation

**TBI**

|  | **Session Count** | | | | | | | | | | | |
| --- | --- | --- | --- | --- | --- | --- | --- | --- | --- | --- | --- | --- |
|  | 1 | 2 | 3 | 4 | 5 | 6 | 7 | 8 | 9 | 10 | 11 | 12 |
| **Walk time** |  |  |  |  |  |  |  |  |  |  |  |  |
| n | 24 | 22 | 24 | 23 | 21 | 15 | 16 | 15 | 13 | 10 | 8 | 7 |
| mean ± sd | 6:30 ± 4:31 | 12:02 ± 5:12 | 12:47 ± 5:20 | 14:13 ± 5:09 | 14:39 ± 5:27 | 16:51 ± 6:07 | 14:48 ± 5:18 | 15:55 ± 7:11 | 15:28 ± 7:39 | 10:19 ± 7:51 | 11:36 ± 4:21 | 13:10 ± 6:40 |
| **Up Time** |  |  |  |  |  |  |  |  |  |  |  |  |
| n | 25 | 24 | 24 | 23 | 21 | 16 | 16 | 15 | 13 | 10 | 8 | 7 |
| mean ± sd | 13:30 ± 6:59 | 18:17 ± 5:36 | 17:47 ± 6:37 | 19:45 ± 5:15 | 19:41 ± 5:04 | 21:14 ± 4:57 | 19:58 ± 5:04 | 19:52 ± 9:04 | 20:02 ± 5:42 | 16:10 ± 9:03 | 15:44 ± 4:01 | 16:42 ± 5:58 |
| **Steps** |  |  |  |  |  |  |  |  |  |  |  |  |
| n | 24 | 22 | 24 | 23 | 21 | 15 | 16 | 15 | 13 | 10 | 8 | 7 |
| mean ± sd | 160.9 ± 123.5 | 310.8 ± 179.2 | 326.4 ± 162.2 | 365.4 ± 160.6 | 379 ± 167.1 | 423.4 ± 284 | 398.3 ± 223.5 | 367.8 ± 287.5 | 438.6 ± 348.1 | 311.1 ± 348.3 | 329.5 ± 224.9 | 466 ± 346.1 |
| **Walk Time to Up Time Ratio** |  |  |  |  |  |  |  |  |  |  |  |  |
| n | 24 | 22 | 24 | 23 | 21 | 15 | 16 | 15 | 13 | 10 | 8 | 7 |
| mean ± sd | 0.44 ± 0.2 | 0.63 ± 0.2 | 0.71 ± 0.17 | 0.72 ± 0.18 | 0.72 ± 0.17 | 0.78 ± 0.16 | 0.73 ± 0.13 | 0.62 ± 0.22 | 0.75 ± 0.2 | 0.65 ± 0.26 | 0.72 ± 0.1 | 0.76 ± 0.11 |
| **Steps to Up Time Ratio** |  |  |  |  |  |  |  |  |  |  |  |  |
| n | 24 | 22 | 24 | 23 | 21 | 15 | 16 | 15 | 13 | 10 | 8 | 7 |
| mean ± sd | 0.18 ± 0.11 | 0.27 ± 0.12 | 0.3 ± 0.09 | 0.31 ± 0.12 | 0.31 ± 0.11 | 0.32 ± 0.16 | 0.32 ± 0.13 | 0.28 ± 0.15 | 0.34 ± 0.17 | 0.31 ± 0.2 | 0.33 ± 0.15 | 0.45 ± 0.24 |

sd = standard deviation
